# Molecular Effects of Polymorphism in the 3’UTR of *Unc-5 homolog C* Associated with Conception Rate in Holsteins

**DOI:** 10.1371/journal.pone.0131283

**Published:** 2015-07-06

**Authors:** Mayumi Sugimoto, Yusaku Gotoh, Takayoshi Kawahara, Yoshikazu Sugimoto

**Affiliations:** 1 National Livestock Breeding Center, Nishigo, Fukushima, Japan; 2 Holstein Cattle Association of Japan, Hokkaido Branch, Sapporo, Hokkaido, Japan; 3 Shirakawa Institute of Animal Genetics, Nishigo, Fukushima, Japan; University of Bologna, ITALY

## Abstract

Conception rates among dairy cows in Japan have declined in recent decades. To enhance our understanding of the genes involved in conception rates, we conducted a genome-wide association study (GWAS) using 822 Holsteins and identified a single-nucleotide polymorphism (SNP) associated with conception rate: A+169G in the 3’ untranslated region (UTR) of *unc-5 homolog C* (*UNC5C*). Cows with higher conception rates carried the A polymorphism in the *UNC5C* 3’UTR. Luciferase assays and quantitative analysis of allele ratios revealed that *UNC5C* transcripts with the A polymorphism were expressed at higher levels than those carrying the G polymorphism. UNC5C transmits either pro- or anti-apoptotic signals depending on the availability of its ligand, Netrin-1. UNC5C expression is negatively regulated by reproductive homeobox X-linked 5 (Rhox5), and the Rhox5 locus is methylated by G9a methyltransferase. G9a-knockout mice have previously been demonstrated to be subfertile, and we found that UNC5C, G9a, and Netrin-1 expression levels increased from the 4-cell stage to the blastocyst stage in fertilized murine embryos, whereas Rhox5 expression decreased. Repression of UNC5C, G9a, or Netrin-1 or forced expression of Rhox5 in the anterior nucleus stage inhibited development to the blastocyst stage, suggesting that cows carrying the G polymorphism in *UNC5C* might have lower conception rates because of the poor development of preimplantation embryos. This study provides novel insights into the role of UNC5C during embryonic development.

## Introduction

Conception rates have decreased dramatically over recent decades in Japanese dairy industry. Means of first service conception rates on 1990 to 2007 decreased from 52.3% to 42.2% [1]. To identify genetic factors affecting variation in conception rate, several genome-wide association studies (GWAS) have been conducted [2–9], but few responsible genes have been identified [10–12]. Here we report a new gene, *unc-5 homolog C* (*UNC5C*), which is associated with conception rate in the Holstein cattle population.

UNC5C is a proapoptotic molecule that governs axon migration in cooperation with its ligand, netrin-1 [13, 14]. Although UNC5C has been well studied in the brain, its expression has recently been determined to be controlled by reproductive homeobox X-linked 5 (Rhox5) in Sertoli cells in the testis, and *UNC5C* mutant mice have been shown to possess decreased male germ cell apoptosis [15], suggesting that the protein functions in the reproductive tract.

Rhox5 belongs to the reproductive homeobox gene cluster on the X chromosome and regulates reproductive processes [16]. Because Rhox5 has been detected in the ovary [17], it is possible that UNC5C may also function in the female reproductive tract under the control of Rhox5.

Rhox5 expression is controlled by DNA methylation [18], which requires histone methylase G9a [19]. Interestingly, G9a-knockout female mice are subfertile [20]. These results imply that the G9a-Rhox5-UNC5C pathway might be important for female fertility.

Here, we report that UNC5C is a novel locus associated with conception rate in Holsteins. We also found that UNC5C, Rhox5, and G9a were selectively expressed during embryonic development. Moreover, repression of *UNC5C* or *G9a* or forced expression of *Rhox5* in the anterior nucleus stage inhibited development to the blastocyst stage. Our work has revealed an unexpected role of UNC5C in the female reproductive system.

## Materials and Methods

### Data Availability Statement

The authors confirm that all data underlying the findings are fully available without restriction. All relevant data are within the paper and its Supporting Information file.

### Ethics Statement

All animal experimentation was performed with the approval of the National Livestock Breeding Center Committee on Animal Research (H26-6).

### Samples

**Fig 1:**
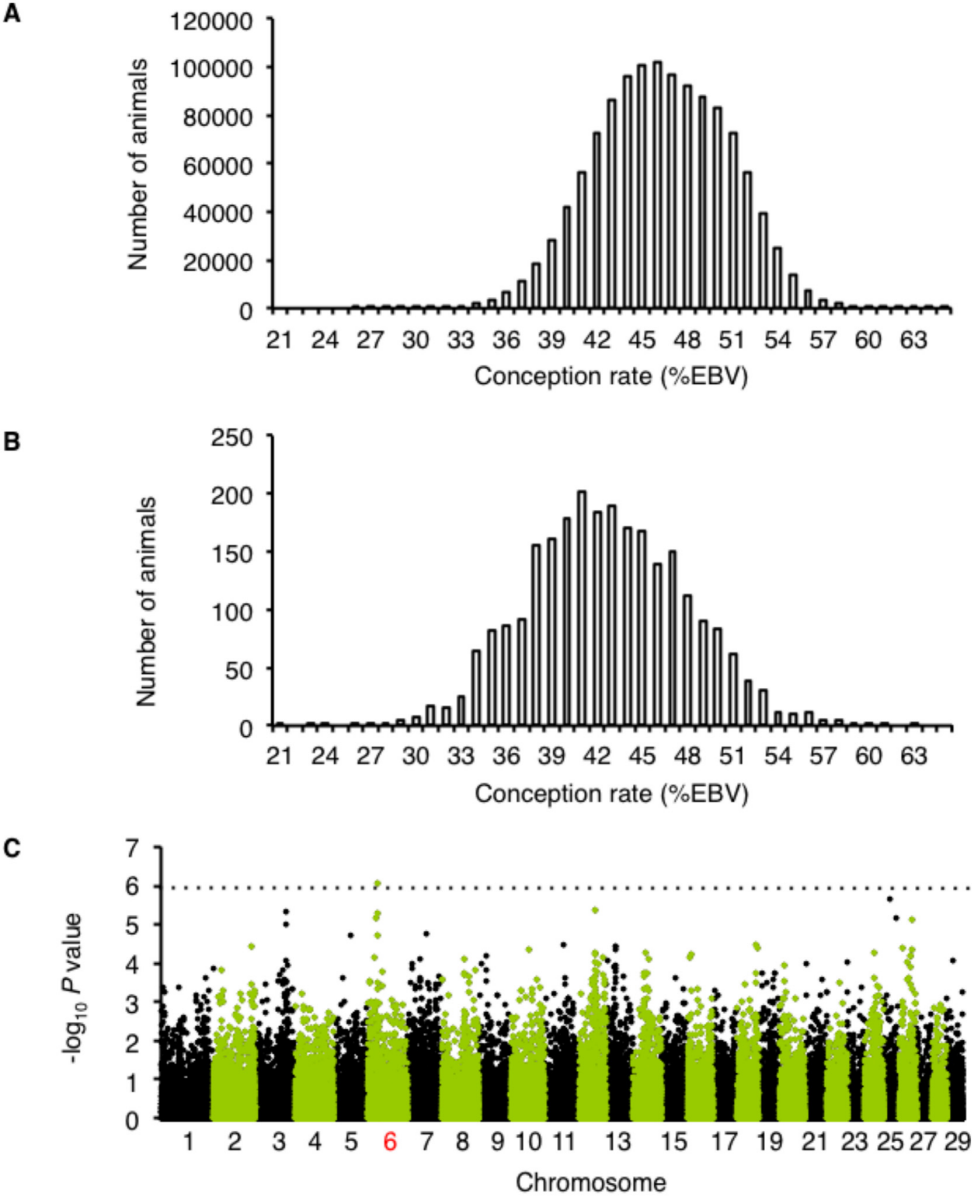
Conception rate is associated with a locus on chromosome 6. A. The distribution of conception rate among cows (females). B. The distribution of conception rate among sires. C. Manhattan plots for the genome-wide screen for loci associated with conception rate. The dotted line represents the threshold for a genome-wide significance of *P* < 1.2×10^-6^ based on the Bonferroni correction for multiple comparisons.

We collected DNA from 2,529 Holstein sires and evaluated the estimated breeding values (EBVs) for the conception rates [1, 21]. The EBV is a genetic component obtained by subtracting an environmental component from a phenotype. The EBVs for the conception rates of the sires were evaluated based on their daughters’ conception rates. The EBVs for conception rates of daughters were evaluated by threshold linear models using insemination event data after first calving. The model can be written as:

l=Xβ+Whh+Wss+Zaa+Zpp+e

where l is a vector of unobserved liabilities; *β* is the vector of systematic effects (herd-year of insemination, month of insemination, days from calving to insemination, regression coefficients on inbreeding, calving ease score of first calving, herd size, and milk yield of the first lactation); h is vector of herd, age, season of insemination; s is vector of service sires; a is vector of additive breeding values; p is vector of permanent environmental effects; e is the vector of residual terms; and X, W_h_, W_s_, Z_a_, and Z_p_ are known incidence matrices with the appropriate dimensions. The mean EBV for the conception rates of the cows was 46.3%. Cows lower than 41.8% belong to the 15^th^ percentile of this population while cows higher than 51% belong to the 85^th^ percentile (Fig 1A). We selected 646 samples with low conception rates (lower than 41.8%) and 176 with high rates (higher than 51%) among the 2,529 sampled sires. We also confirmed that the selected 646 samples had appropriate deregressed EBV (dEBV) because the reliability of specific animal EBVs differs markedly. The dEBV was calculated as follows:

dEBV = (EBV−base)/reliability + base

### Whole-Genome Scan

Selected 822 (= 646+176) samples were genotyped for a total of 54,001 single nucleotide polymorphisms (SNPs) using a Bovine SNP 50 v1 DNA Analysis Kit (Illumina, San Diego, CA, USA) and were adjusted for population stratification by principal component analysis [22] (lamda = 1.2). Logistic regression analysis was conducted using PLINK software [23]. The threshold for the association study was *P* < 1.2×10^-6^ based on the Bonferroni correction. P-values were adjusted for multiple testing using the Bonferroni approach based on 43,852 hypotheses (43,852 SNPs remained after quality control exclusions), with the final adjusted alpha level of 0.05. Conditional logistic regression analysis was performed by including UNC5C (A+169G) as a covariate.

### Identification of Novel SNPs

Each of the exons, 2 kb of the 5’ untranslated regions (UTRs), and 2 kb of the 3’UTRs of genes located in the associated regions based on the Nov. 2009 *Bos taurus* draft assembly [24] (UMD_3.1) were amplified by polymerase chain reaction (PCR) and sequenced. Regions including the genome-wide significant SNPs and their neighboring SNPs with r^2^ values greater than 0.2 were defined as the associated regions. r^2^ values were calculated by a linkage disequilibrium analysis using PLINK software [23]. The primers for each gene and the samples used for comparing the sequences are shown in Tables A and B in S1 File, respectively. We selected four samples each with homozygous high- or low-specific haplotypes comprising the genome-wide significant SNPs and their neighboring SNPs. The haplotypes were constructed manually using homozygous SNPs as shown in Table B in S1 File.

### Genotyping of Additional Samples

DNA was extracted from blood samples drawn from 2,030 cows and their dEBVs for the conception rates were calculated as described before [1, 21]. Genotyping UNC5C (A+169G) of additional 2,030 cows and total 2,529 sires (Bovine SNP 50 v1 DNA Analysis Kit-genotyped 822 sires plus additional 1,707 sires) was performed following PCR amplification. The average conception rate ± SE values for the typed sires and cows were compared. The *p*-value was calculated using Student’s t-test.

### Real-Time PCR

RNA was extracted from bovine brain, heart, kidney, liver, lung, ovary, pancreas, skeletal muscle, and uterus one each using TRIzol reagent (Life Technologies, Carlsbad, CA, USA). Real-time PCR was conducted with an ABI 7900HT Sequence Detection System using the comparative Ct method and glyceraldehyde-3-phosphate dehydrogenase (GAPD) as an internal control (Life Technologies). The primers used in these assays are shown in Table C in S1 File.

### Luciferase Assay

Fragments of the 3’UTR of *UNC5C* were generated using PCR with the respective forward and reverse primers (Table D in S1 File). These PCR products were further amplified via PCR using the forward2 and reverse2 primers (Table D in S1 File) to generate a restriction site. The resulting fragments were then cloned into a pMIR-REPORT miRNA Expression Reporter Vector (Life Technologies). Luciferase assays were performed using a Dual-Luciferase Reporter Assay System (Promega, Madison, WI, USA). For co-transfection with Rhox5, fragments of the 5’UTR of *UNC5C* were generated using PCR with the 5’forward and 5’reverse primers (Table D in S1 File). These PCR products were further amplified via PCR with the 5’forward2 and 5’reverse2 primers (Table D in S1 File) for cloning using an In-Fusion Advantage PCR Cloning Kit (Takara Bio Inc., Shiga, Japan).

### SNaPshot and Quantitative Analysis of Allele Ratios

The allelic messenger RNA (mRNA) ratio was determined using a SNaPshot Multiplex Kit (Life Technologies), and the primers used are shown in Table E in S1 File. For cDNA preparations, each mRNA was converted to cDNA in three separate experiments.

### Generation of Overexpression Construct

We generated murine *Rhox5* using PCR with the respective forward and reverse primers (Table F in S1 File). These PCR products were further amplified via PCR with the forward2 and reverse2 primers (Table F in S1 File) for cloning into a pCAGGS (N-R) vector [25] using an In-Fusion Advantage PCR Cloning Kit (Takara Bio Inc.).

### Immunoblotting Analysis

Protein was extracted from transfected cells using a NucleoSpin RNA/Protein kit (Machrey-Nagel, Düren, Germany). The extracted proteins were separated, transferred to a membrane, and blocked. The blots were incubated with a primary antibody (Table G in S1 File) and detected with ECL Prime (GE Healthcare, Buckinghamshire, UK).

### Generation of siRNA Constructs

Candidate siRNA targets for *UNC5C*, *G9a*, and *Netrin-1* were designed using the GenScript software [26] (Table H in S1 File). These oligonucleotides were annealed, cloned into pSilencer 3.0-H1 (Life Technologies), and transfected into OV2944-HM-1 cells. After transfection, the RNA was subjected to real-time PCR to allow for a comparison of the effects of each candidate siRNA target. The best target for each gene was selected, and a negative control for the corresponding sequence was designed (Table H in S1 File).

### Methylation-Sensitive PCR

DNA was extracted from transfected cells using TRIzol reagent (Life Technologies). Five micrograms of genomic DNA was digested with either the MspI or HspaII restriction enzymes and was PCR-amplified with primers flanking HpaII restriction sites in the *Rhox5* promoter (Table I in S1 File).

### Immunofluorescence Staining of Embryos

Fertilized embryos in the anterior nucleus stage of ICR mice were obtained from CLEA Japan, Inc. (Tokyo, Japan). Frozen embryos were recovered using pre-warmed 0.25 M sucrose (ARK Resource, Kumamoto, Japan), transferred to pre-warmed droplets of KSOM/AA (ARK Resource), and incubated at 37°C in a 5% CO_2_ humidified chamber for 24, 48, 72, and 96 h. The incubated embryos were fixed in 4% formaldehyde and permeabilized with 0.5% Triton X-100. After blocking, the cells were incubated with a primary antibody (Table G in S1 File) and visualized with an IX81 microscope (Olympus, Tokyo, Japan).

### Electroporation

A pmCherry vector (Clontech, Mountain View, CA, USA) was transferred into a pCAGGS (N-R) plasmid [25] to produce protein under the control of a strong ubiquitous promoter based on the β-actin promoter. Fertilized embryos in the anterior nucleus stage were subjected to electroporation in 30 microL of HBS buffer [20 mM HEPES, pH 7.0–7.6 (Sigma-Aldrich, Saint Louis, MO, USA) and 150 mM NaCl] containing 45 microg of mCherry, siUNC5C with mCherry (ratio of 10:1), Rhox5 with mCherry, siG9a with mCherry, or siNetrin-1 with mCherry. siUNC5C and siNetrin-1 were generated and selected as shown in Table H in S1 File. Three sets of four electric pulses (21 V, duration of 1 ms, interval of 99 ms) were delivered using a CUY21SC electroporator (Nepagene, Chiba, Japan) [27].

## Results

To identify genes associated with fertility, we selected 646 samples with low conception rates (lower than 41.8%) and 176 with high rates (higher than 51%) among the 2,529 sampled sires (Fig 1B). Based on the typing of a total of 822 samples for 54,001 SNPs, we identified a novel locus associated with conception rates on chromosome 6 (Fig 1C).

**Fig 2:**
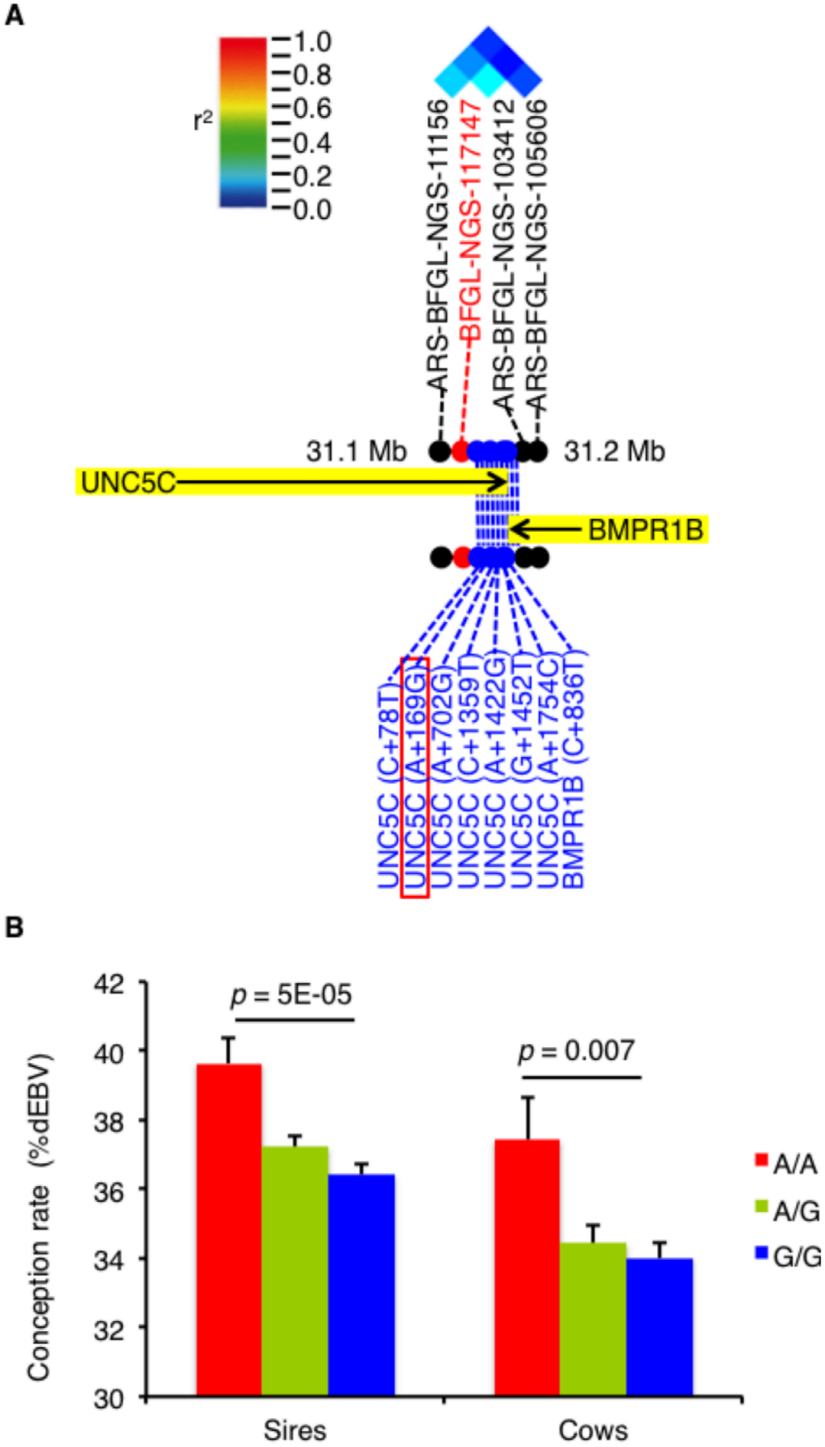
The *UNC5C* 3’UTR SNP is associated with conception rate. A. A pairwise linkage disequilibrium diagram showing a schematic representation of the genes (black arrow) located in the associated region on chromosome 6. The red, black, and blue dots represent the genome-wide significant SNPs, the original SNPs, and the newly developed SNPs, respectively. B. The average conception rate ± SE values for the sires and cows in terms of dEBV. The *p*-value was calculated using Student’s t-test.

The associated region on chromosome 6 harboring the significant SNP, BFGL-NGS-117147, included *UNC5C* and *bone morphogenetic protein receptor type IB* (*BMPR1B*; Fig 2A). To detect possible causative polymorphisms in this region, we sequenced all exons and the 5’ and 3’UTRs of these genes and found 8 novel SNPs (Fig 2A). Reanalysis of the newly developed SNPs demonstrated that UNC5C (A+169G) was the most significant (Table J in S1 File). We also confirmed that the association signal for BFGL-NGS-117147 disappeared when the UNC5C (A+169G) genotype was included as a covariate in logistic regression analysis (*p* = 0.02). Moreover, we genotyped UNC5C (A+169G) in an additional 2,030 cows and total 2,529 sires and found that cattle harboring the A/A genotype exhibited higher conception rates than those harboring the G/G genotype (Fig 2B). Thus, UNC5C (A+169G) was the most promising causative SNP on chromosome 6.

**Fig 3:**
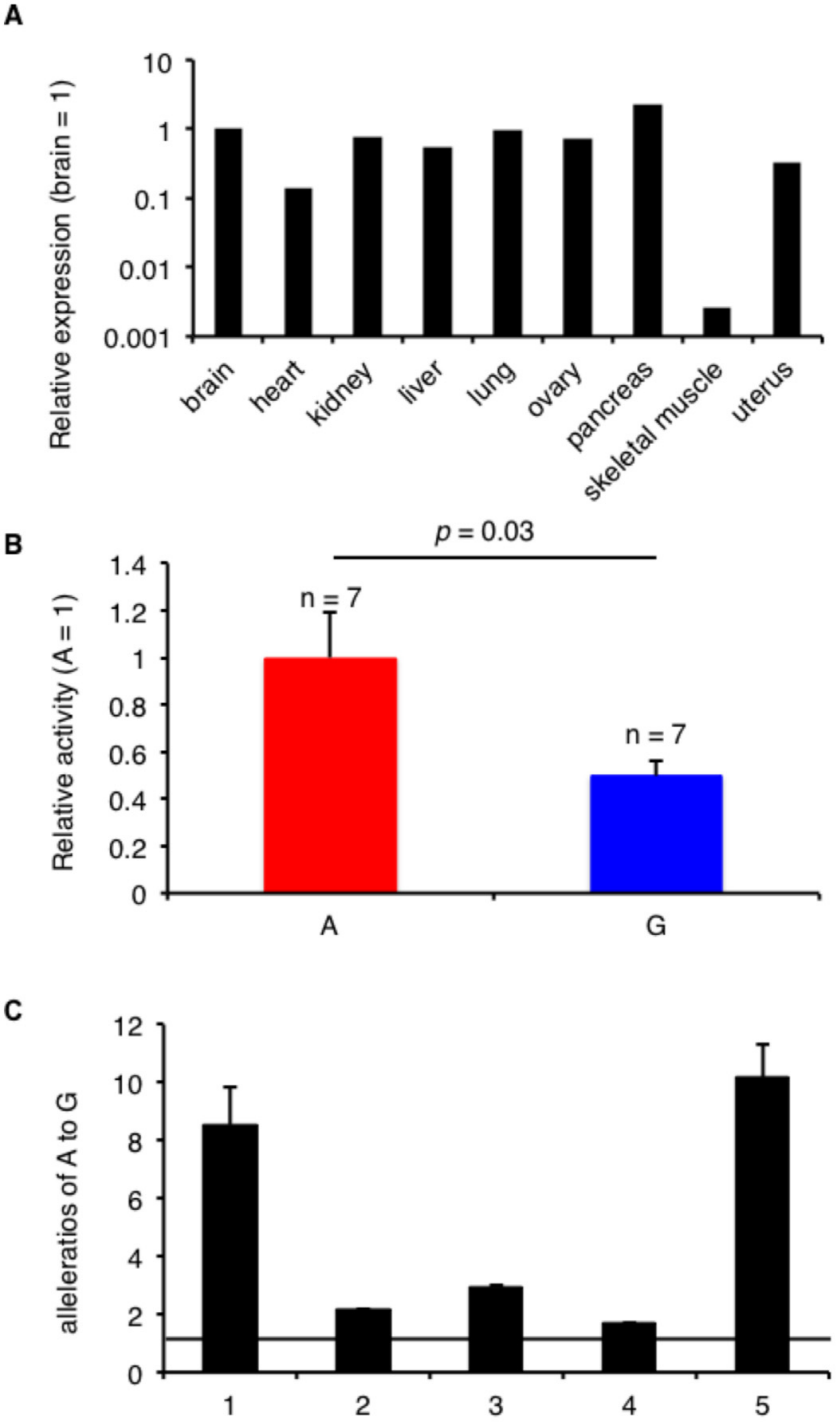
The *UNC5C* 3’UTR SNP controls its expression level. A. *UNC5C* expression levels in bovine tissues, as determined via real-time PCR. B. The relative luciferase activity of the 3’UTR region of *UNC5C* in OV2944-HM-1. The data are presented as the means ± SEM. The *p*-value was calculated using Student’s t-test. C. The average allele-specific mRNA expression level of *UNC5C* ± SEM in the heterozygous bovine ovaries based on SNaPshot (n = 5). The ratios of A to G relative to the genomic DNA are shown.

UNC5C (A+169G) is located in the 3’UTR of *UNC5C* and may influence the expression level of this gene. Because *UNC5C* is expressed in several bovine tissues, including the ovary (Fig 3A), we used the murine ovarian tumor line OV2944-HM-1 to assess luciferase activity. Reporters carrying the A allele exhibited higher enzyme activity than those carrying the G allele (Fig 3B). Consistent with the results of the luciferase assay, the level of mRNA generated in the presence of the A allele was higher than that produced in the presence of the G allele according to the allelic mRNA ratio measured in the bovine ovaries (Fig 3C). Consequently, the *UNC5C* expression level might affect conception rate in cattle.

**Fig 4:**
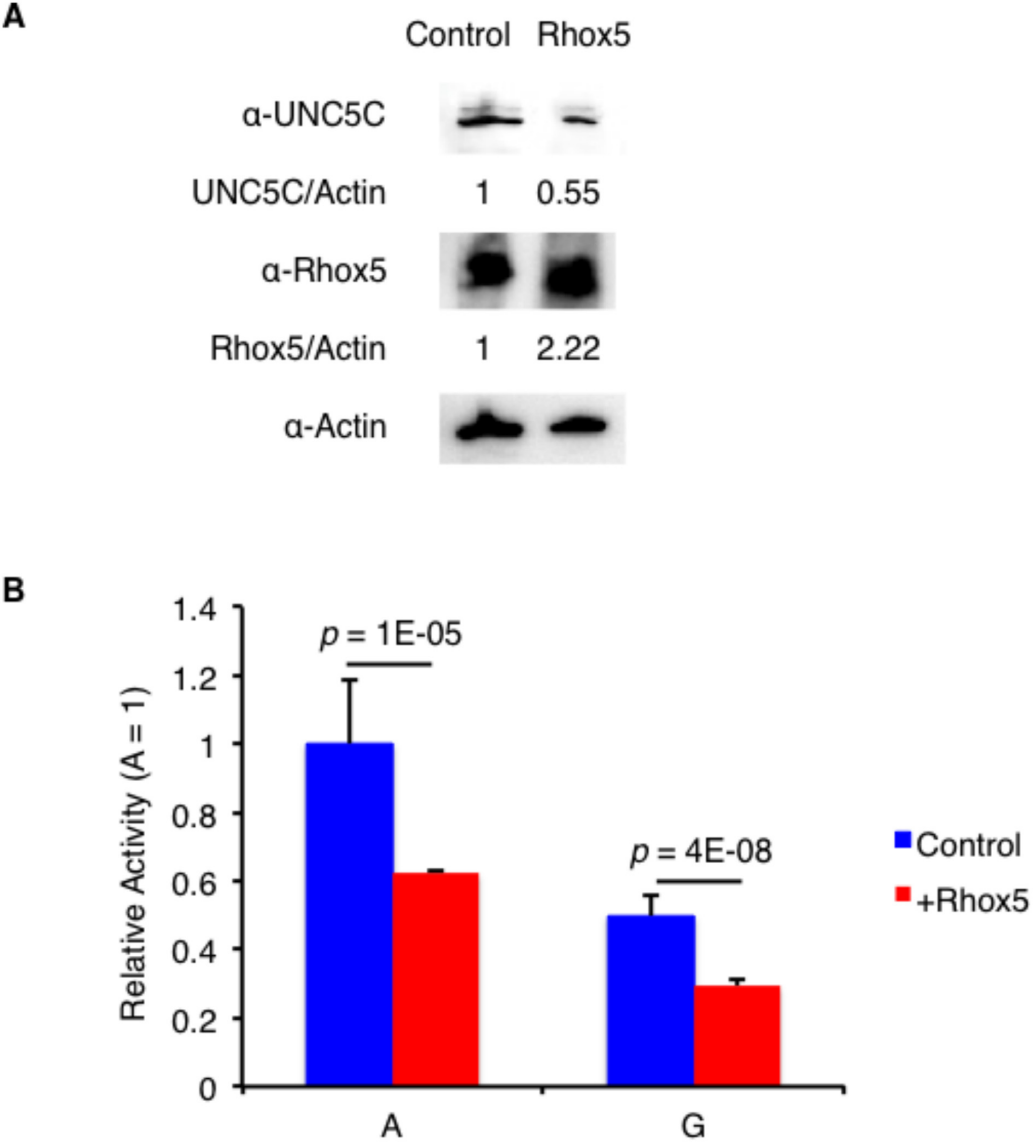
Rhox5 decreases UNC5C expression. A. Representative immunoblots with anti-UNC5C, anti-Rhox5, and anti-actin antibodies for OV2944-HM-1 transfected with vector (Control) or Rhox5. B. The relative luciferase activity at the 3’UTR of *UNC5C* in OV2944-HM-1 co-transfected with vector (Control) or Rhox5. The data are presented as the means ± SEM (n = 6). The *p*-value was calculated using Student’s t-test.

UNC5C is a proapoptotic molecule [13], and Rhox5 controls its expression in Sertoli cells in the murine testis [20]. To examine whether the Rhox5 expression level affected the UNC5C concentration in the murine ovary, we transfected OV2944-HM-1 with an empty vector or Rhox5 (a *Rhox5* expression plasmid). As shown in Fig 4A, increased Rhox5 expression decreased UNC5C expression. Moreover, co-transfection of reporters covering both the 5’ and 3’UTRs of *UNC5C* with an empty vector or Rhox5 revealed that increased expression of Rhox5 decreased the luciferase activities of both reporters carrying the A and G alleles at the 3’UTR (Fig 4B), suggesting that its expression may affect that of UNC5C.

**Fig 5:**
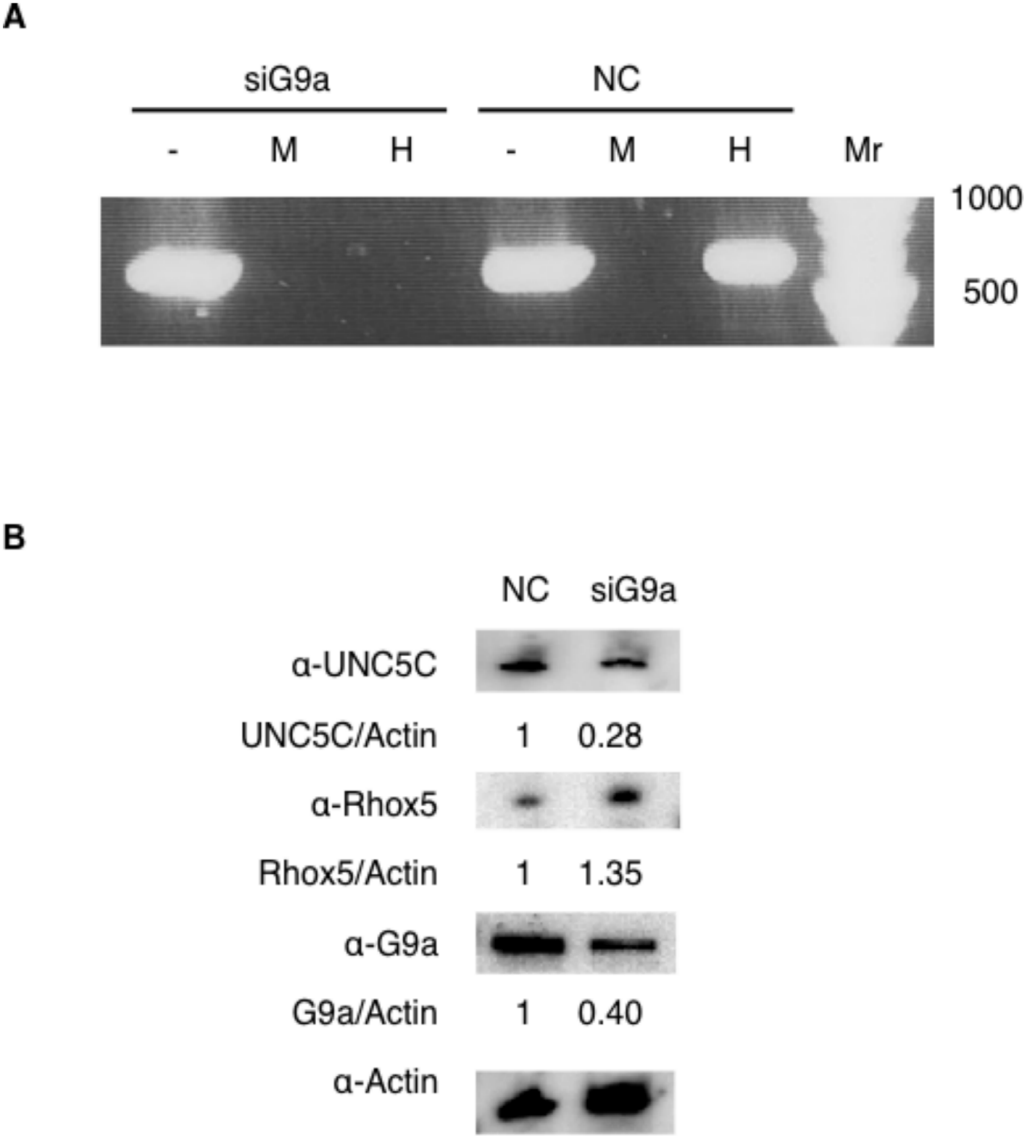
G9a decreases Rhox5 expression. A. DNA methylation at the *Rhox5* promoter as assessed by PCR following digestion with the methylation-insensitive MspI (M) or methylation-sensitive HpaII (H) restriction enzymes of the genomic DNA from OV2944-HM-1 transfected with siG9a or negative control (NC). “-”represents undigested DNA. “Mr” is a DNA size marker. B. Representative immunoblots with anti-UNC5C, anti-Rhox5, anti-G9a, and anti-actin antibodies for OV2944-HM-1 transfected with negative control (NC) or siG9a.

Because Rhox5 expression is controlled by DNA methylation [18] and the methylation of Rhox loci requires the histone methylase G9a in mouse embryonic fibroblasts [19], the level of G9a expression might influence methylation of the Rhox5 locus in the murine ovary. To explore this possibility, we performed methylation-sensitive PCR to assess murine OV2944-HM-1 transfected with siG9a, which represses the expression of endogenous *g9a* in mice, or that transfected with a negative control. We found that the Rhox5 locus was hypomethylated in the siG9a-transfected cells (Fig 5A). Furthermore, immunoblotting of OV2944-HM-1 transfected with siG9a revealed that decreased G9a expression increased Rhox5 expression and decreased UNC5C expression (Fig 5B). Therefore, these results imply that G9a expression may affect UNC5C expression through Rhox5 methylation.

**Fig 6:**
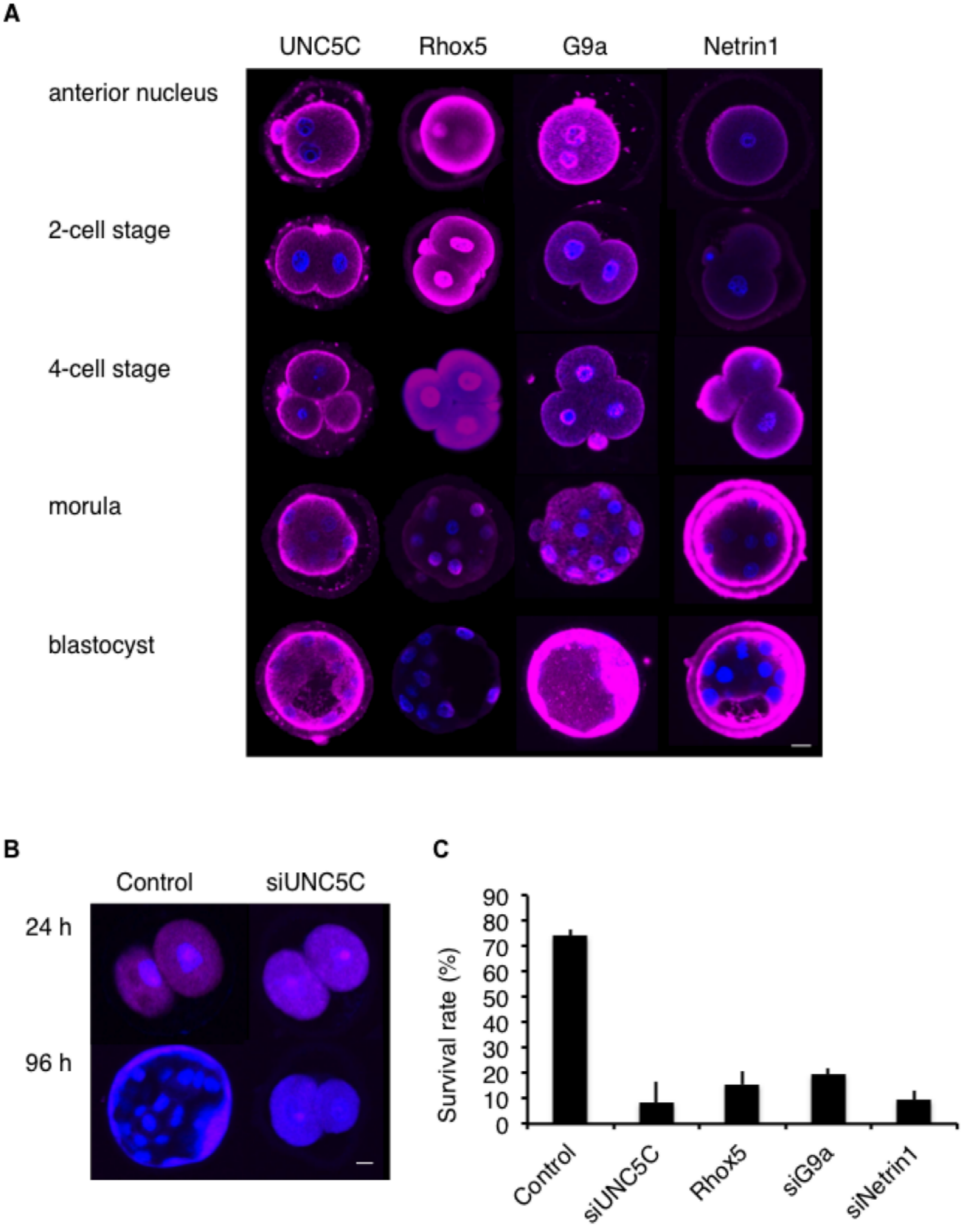
G9a decreases Rhox5 expression. A. Representative images showing UNC5C, Rhox5, G9a, and Netrin-1 (each shown by pink fluorescence) in the anterior nucleus, 2-cell, 4-cell, morula, and blastocyst stages. Bar: 10 μm. B. Representative images showing mCherry (pink fluorescence) in murine embryos at 24 or 96 h after electroporation with mCherry only (Control) or with mCherry and siUNC5C (siUNC5C). Bar: 10 μm. C. Survival rates of murine embryos at 96 h after electroporation with mCherry only (Control), mCherry and siUNC5C (siUNC5C), mCherry and Rhox5 (Rhox5), mCherry and siG9a (siG9a) or mCherry and siNetrin-1 (siNetrin-1).

Because G9a-knockout female mice are subfertile [20], G9a, Rhox5, and UNC5C expression might affect fertility. To explore this possibility, we first assessed G9a, Rhox5, and UNC5C expression in fertilized murine embryos. As shown in Fig 6A, the expression levels of UNC5C and G9a increased from the 4-cell stage to the blastocyst stage. However, Rhox5 expression decreased from the 4-cell stage to the blastocyst stage, whereas the expression of Netrin-1, which is a ligand of UNC5C, increased. These distinct expression patterns suggest that the G9a-Rhox5-UNC5C pathway might be important for embryonic development.

To further evaluate the effects of UNC5C on embryonic development, we examined murine embryos after transferring siUNC5C to the embryo in the anterior nucleus stage with a control mCherry vector using electroporation. At 24 h after electroporation, we confirmed the expression of mCherry, and embryonic development proceeded to the 2-cell stage (Fig 6B), indicating that the transfer of the external DNA was successful and that the embryos were surviving. However, at 96 h after electroporation, only those embryos electroporated with the control became blastocysts (Fig 6B). The survival rates at 96 h after the electroporation of siUNC5C, Rhox5, siG9a, and siNetrin-1 were lower than those of the control (Fig 6C). Consequently, G9a, Rhox5, UNC5C, and Netrin-1 expression might be important for embryonic development, and SNPs in bovine *UNC5C* might affect conception rates by influencing this process.

## Discussion

The GWAS described here detected a significant SNP in *UNC5C* associated with conception rate (*P* = 5.6×10^-9^, Table SJ in S1 File). *UNC5C* locates at 31 Mb on chromosome 6 (Fig 2A). Interestingly, luteinizing hormone (LH) level has been associated with the similar region on BTA6 [28]. They measured plasma levels of LH after a gonadotropin-releasing hormone challenge in young cattle and conducted GWAS. Because plasma levels of LH were correlated to age at puberty [29], *UNC5C* might affect age at puberty through germ cell development. In fact, age at puberty itself has been mapped at 29–30 Mb on chromosome 6 [30]. Interval to first estrus after calving has also been associated at 34 Mb on this chromosome [2], implying that *UNC5C* might influence the control of estrus cycle as well as conception rate.

A limitation of GWAS relates to sample size, however, our sample size is powered to identify associations greater with a population (control) allele frequency of 0.2, at p = 0.01 significance level with 80% power (calculated using the Genetic Power Calculator [31]). Among 2529 sampled sires, we selected 646 samples with low conception rates belonging to the 15^th^ percentile of the population and 176 samples with higher rates belonging to the 85^th^ percentile for GWAS. Comparing samples carrying two extreme scores could increase power of calculation.

*UNC5C* encodes a proapoptotic molecule [13]. In mice, UNC5C controlled male germ cell apoptosis in the testis [15]. Our data suggested that UNC5C was associated with conception rate through embryonic development in cattle. In humans, both male and female fertilities were associated with genes related with apoptosis. Family size in the Hutterite sample was associated with ubiquitin-related peptidase 8 [32], which affects apoptosis and assembles acrosomes in differentiating sperm cells [33]. Khan et al. conducted genome-wide expression analysis in endometriosis women and revealed that expression of genes in pathways directly and indirectly associated with cell apoptosis and survival were differentially affected [34]. Therefore, genes related with apoptosis might have important roles in reproductive process in mammals.

Luciferase assays and quantitative analysis of the allele ratios revealed that the identified SNP in the 3’UTR affected *UNC5C* expression. Polymorphisms in 3’UTRs may affect the binding of microRNAs, which regulate mRNA and protein expression levels [35]. Although we could not find a candidate microRNA that bound to the SNP region at the 3’UTR of *UNC5C* using available software, it is possible that an unknown microRNA might affect its expression via the SNP UNC5C (A+169G).

The methylation-sensitive PCR and immunoblotting assays indicated that Rhox5 and G9a affect UNC5C expression. Moreover, the electroporation experiments suggested that UNC5C, Rhox5, and G9a expression might have influenced the development of the fertilized embryos. Tachibana et al. have reported that fertilized maternally G9a-deprived embryos do not reach the blastocyst stage [36]. An additional methyltransferase, ESET, is required for the appropriate development of the inner cell mass of the blastocyst [37]. Methyltransferases and their targets might be important for preimplantation embryonic development.

The manner by which UNC5C controls embryonic development is unknown. UNC5C alone promotes apoptosis; however, it also promotes cell growth in the presence of its ligand, Netrin-1 [38]. Our immunofluorescence staining experiment showed that UNC5C and Netrin-1 expression levels increased from the 4-cell stage to the blastocyst stage (Fig 6A). UNC5C, together with Netrin-1, might mediate embryonic growth.

In conclusion, the present work investigated 54,001 SNPs covering whole genome in 646 samples with low conception rates and 176 samples with high conception rates. Our analysis using well-estimated phenotype, dEBV, showed a significant association of a SNP in the 3’UTR of UNC5C in conception rate in dairy cattle. Further functional studies revealed that the SNP in the 3’UTR of UNC5C related the expression level of this gene, which affected the development of preimplantatin embryos. These results have suggested that UNC5C may play a role in embryonic development, providing a foundation for understanding genes involved in fertility.

## Supporting Information

S1 File 10 Tables containing primers and samples’ information. Primers used to search for SNPs (Table A). Samples used for developing new SNPs (Table B). Primers used for real-time PCR (Table C). Primers used for generating reporter constructs (Table D). Primers used for SNaPshot (Table E). Primers used for generating overexpression constructs (Table F). Antibodies used in this study (Table G). Oligonucleotides used for generating siRNA constructs (Table H). Primers used for methylation-sensitive PCR (Table I). Association signals for the locus that correlated with conception rate in Holsteins (Table J).
(XLS)
